# COVID-19 should be a novel indication for fertility preservation

**DOI:** 10.5935/1518-0557.20200048

**Published:** 2020

**Authors:** Selmo Geber, Natalia Prates, Marcos Sampaio, Marcello Valle, Marcos Meseguer

**Affiliations:** 1 Department of Obstetrics and Gynaecology of the Universidade Federal de Minas Gerais, Brazil; 2 ORIGEN, Center for Reproductive Medicine, Brazil; 3 Instituto Valenciano de Infertilidad, Universidad de Valencia, Valencia, Spain

On March 11th, 2020, the World Health Organization (WHO) declared the coronavirus disease 2019 (COVID-19) a pandemic. Since then, all federal and local authorities as well as regulatory agencies have asserted and developed robust policies in order to reduce the spread of the disease, worsening of clinical presentation and deaths related to the infection with the severe acute respiratory syndrome coronavirus 2 (SARS-CoV-2), the virus responsible for COVID-19. Their statements also address concerns on the risks to pregnant women and maternal transmission to babies.

As much as reproductive medicine is concerned, physicians and patients are still searching the best ways to face the challenges that are still ahead. Therefore, it is very likely that all reproductive medicine scientific societies worldwide have published its guidelines to head local authorities and reproductive medicine professionals on what would be the best conduct. Although there is still a lack of scientific evidence on whether there is a negative impact of the COVID-19 on reproductive outcomes as well as vertical transmission, the most frequent suggestions have been to avoid pregnancies and discontinue fertility treatments, with exception for fertility preservation in oncologic patients and urgent cases (ASRMa; ESHREa; REDLARA & SBRA, 2020). These recommendations aimed to reduce non-essential contacts and prevent possible maternal and fetal complications and also to support the necessary reallocation of healthcare resources.

More recently, new orientations have been published to guide Assisted Reproductive Technology (ART) treatments restart for any clinical indication, where COVID-19 infection is decreasing, in line with local regulations (ASRM, 2020b; ESHRE, 2020b). However, so far, we do not know how long this pandemic will last, and when life will return to normal. Due to this uncertainty, we also cannot affirm when clinics will be able to offer routine basis treatment for all infertile couples looking for treatment. Postponing ART cycles, might represent disastrous consequences for older patients, those with reduced ovarian reserve and also for those with financial problems. In this group of patients, the restriction for ART treatments might affect the chance to conceive after the end of this period.

According to the WHO, infertility is a disease of the reproductive system which generates disability and affects more than 50 million couples around the world (Mascarenhas *et al.*, 2012). A considerable amount of this population has benefited from ART as it has been estimated that over 8 million babies have been born since the birth of the first IVF baby. Not to mention that more than a half million babies that are born from over 2 million in vitro fertilization cycles per year (Fauser, 2019). Infertility treatment changes and cancellations are creating a clear crisis for those who badly want to have a child. Thus, it should be relevant if scientific organizations, governments and regulatory health agencies could consider the inclusion of fertility preservation treatment in the forthcoming guidelines in order to minimize the negative impact of delaying ART treatment on pregnancy rates.

Fertility preservation might be performed through gamete and embryo cryostorage. Sperm cryopreservation is and old and well stablished technique that have been showing regular results for more than fifty years. Embryo freezing is also a well stablished technique, being performed for over 30 years. Moreover, recently, it has been reported that the transfer of frozen/thawed embryos provide similar or even better results than those observed after fresh cycle embryo transfer (Roque *et al*., 2019).

Oocyte cryopreservation is no longer an experimental technique and is considered one of the most important advances in assisted reproduction. It is indicated for oncologic patients and for those who decide to postpone motherhood. The results are comparable to those observed with embryo freezing proving to be effective for protecting fertility (Cobo *et al.*, 2018). However, even for fertility preservation, age and ovarian reserve are fundamental for success since the more oocytes one gets, the higher the pregnancy rates. These facts reiterate the need not to impose a delay in infertility treatment and therefore fertility preservation should be offered for all patients who cannot or are not allowed to become pregnant at this moment.

Moreover, it is indeed fundamental to consider that ART treatment and fertility preservation do not implicate any harm to the patients, as, so far, there have been no confirmation of transmission of COVID-19 via gametes and embryos, and there is no scientific evidence of uterine vertical transmission (Cochrane Gynaecology and Fertility, 2020). Also, as we do not know how long this pandemic will last and when life will return to normal, irrespective of all efforts that have been made worldwide, instead of not doing nothing and, as doing nothing, we can worsen patient’s reproductive prognosis, we could preserve fertility and, at least, postpone our doubts and uncertainties.

Accordingly, it is our opinion that COVID-19 could be a new indication for fertility preservation for a determined deadline, until complete return of assisted reproduction treatments in regular basis.
